# Decline in hospitalization for genital warts in the Veneto region after an HPV vaccination program: an observational study

**DOI:** 10.1186/s12879-017-2361-5

**Published:** 2017-04-05

**Authors:** Silvia Cocchio, Tatjana Baldovin, Chiara Bertoncello, Alessandra Buja, Patrizia Furlan, Mario Saia, Vincenzo Baldo

**Affiliations:** 1 Institute of Hygiene, Laboratory of Public Health and Population Studies, University of Padua, Via Loredan 18, 35121 Padua, Italy; 2 EuroHealth Net, Venice, Veneto Region Health Directorate, Venice, Italy

**Keywords:** HPV, Hospitalization, Genital warts

## Abstract

**Background:**

Human papillomavirus (HPV) is one of the most common sexually transmitted pathogens. This observational study was conducted to estimate the trend of hospitalization for genital warts (GWs) in the Veneto region (Italy) from 2004 to 2015.

**Methods:**

All patients with GWs were identified in the hospital discharge records of all public and accredited private hospitals that related to Veneto residents and contained the ICD9-CM code 078.11 associated with a genital surgical procedure (vulval/vaginal warts, penile warts and anal warts). Annual total and sex- and age-specific hospitalization rates and trends were calculated and correlated with the different HPV vaccine coverage over the study period.

**Results:**

An annual rate of 11.8 per 100,000 population (8.6 per 100,000 males, and 14.8 per 100,000 females) was found, corresponding to 6076 hospitalizations for condyloma (53.3% vulval/vaginal, 35.8% anal, 8.3% penile, and 2.6% both penile or vulval/vaginal and anal). Among females, the rate of overall GWs remained stable to 2007 (19.1 per 100,000), then dropped significantly, reaching a rate of 11.3 per 100,000 in 2015 (average annual percent changes [AAPC]: -6.1%; 95% CI: -8.4; −3.7). For males, the overall rate increased over the study period (from 6.4 per 100,000 in 2004 to 10.8 per 100,000 in 2015; AAPC: 3.8%; 95% CI: 1.2; 6.4).

Among the potentially vaccinated females (12- to 20-year-olds) there was a 62.1% decrease in the number of vulval/vaginal warts from the years 2010-2012 to the years 2013-2015 due to an increase in the HPV coverage rate. A similar reduction among males was observed in the same period and the same age group for penile warts (−68.2%).

**Conclusion:**

GWs have an important impact on the health services and data suggest that GW-related hospitalization rates rapidly decline in a population with a high HPV vaccination coverage (about 75%). Further efforts should be made to better clarify the epidemiological picture regarding HPV-related diseases, with particular regard to sexual behavior.

## Background

Human papillomavirus (HPV) is one of the pathogens most often transmitted sexually in the developed and developing world. Despite they can be associated with malignant lesions, the HPV types generally give rise to benign changes such as genital warts (GWs), which are benign epithelial mucosal tumors. About 90% of diagnosed GWs are associated to HPV types −6 and 11 [1]. GWs are an important public health issue in both sexes, in European regions point to an estimated annual incidence ranging from 142.0 to 191.1 per 100,000 females, and from 147.6 to 167.7 per 100,000 males [2, 3]. The peak incidence is between 20 and 24 years of age for females, and between 25 and 29 years old in males [4]. GWs are not life-threatening and are generally not perceived as a serious condition, but they can have an important impact in the quality of life of the person affected [5].

Hospital discharge records can be useful for assessing the reporting of cases of GW severe enough to warrant admission to hospital [6]. In industrialized countries, the percentages of GW patients that are hospitalized vary between approximatively 7% and 19% [7–9]. Patients are only hospitalized in the more complex cases, mainly for GWs requiring surgical removal [10]. The hospitalization rate for GWs is clearly only the tip of the iceberg of the burden of care for HPV-related diseases, but its quantification is useful for assessing the effectiveness of any prevention strategy.

HPV is highly contagious and barrier measures cannot guarantee full protection. Even sexual intercourse without penetration is considered sufficient for transmission [11]. Only an effective vaccine against HPV can ensure primary prevention of the infection. Two anti-HPV vaccines have been developed and are currently used in population vaccination programs in many countries. Both have proved highly effective in preventing HPV 16 and 18, and the quadrivalent vaccine also protects against the HPV types that cause 90% of GWs (i.e. types 6 and 11) [12, 13].

Since 2008, free anti-HPV vaccination (with a quadrivalent vaccine against HPV 16 and 18, and 6 and 11 [4-HPV]) has been offered to all girls in the Veneto region (Italy) in their 12th year of life, and since 2015 it has also been offered to males of the same age. It remains available free of charge up until their 18th birthday, then vaccination is offered in co-payment to women up to 46 years old and to men up to 26 years old. As at December 2015, the mean vaccination rate against HPV in the Veneto (Italy) was reportedly about 75%, for full vaccination cycles [14].

The aim of the present study was to estimate the trend of GW-related hospitalization rates, stratified by gender, and to assess the impact of HPV vaccination.

## Methods

We conducted this observational study in the Veneto region, which has a population of 4.9 million. We checked the hospital discharge records (HDRs) related to Veneto residents in all public and accredited private hospitals from 1 January 2004 to 31 December 2015, considering the following HDR data: date of birth, gender, place of residence, date of admission, surgical and other procedures, and date of discharge. Each HDR contained one primary (or first-listed) diagnosis, and up to five secondary diagnoses based on the codes of the International Classification of Diseases, Ninth Revision, Clinical Modification (ICD-9-CM).

Hospital discharge records were considered if they contained the ICD9-CM code 078.11 (condyloma acuminatum) in one of the six diagnosis fields. We analyzed only GW-related hospitalizations that included the following ICD9-CM surgical codes grouped by anatomical site: vulval/vaginal warts (ICD9-CM: 70-71 and 58.3), penile warts (ICD9-CM: 64 and 58.3), and anal warts (ICD9-CM: 49). The length of hospital stay was calculated as the days elapsing between the dates of admission and discharge for regular admissions, or the number of admissions for day hospital patients, and the mean hospital stay was calculated. For patients readmitted, only the first hospital stay was considered. Data were obtained from the Regional Statistics Office of Veneto Region.

Based on the total number of hospital admissions concerning Veneto residents in each year considered, specific annual GW-related hospitalization rates were calculated by dividing the annual number of GW-related hospitalizations by the population in the year considered (according to the Veneto Regional Authority’s statistical office), and expressing the rates as hospitalizations per 100,000 population.

Subjects were grouped by age at the time of their hospital admission as follows: 12-20 years old (all women in this age group were eligible for free vaccination at all times during the study period), 21-27 years old, 28-47, and 48+ years old.

The estimated HPV vaccination coverage among 12- to 20-year-olds increased year by year from 2008 onwards. The study period was divided as follows: a) a pre-vaccination period (2004-2006); b) an early HPV vaccination period (2007-2009), when the coverage was still low (about 15% of the 12- to 20-year-old cohort); c) an intermediate HPV vaccination period (2010-2012), when the coverage in this age group reached about 43%; and d) a late HPV vaccination period (2013-2015), when it was about 75%.

It was impossible to estimate the coverage for other birth cohorts.

### Statistical analysis

The data were analyzed using Student’s t-test for continuous data and Pearson’s chi square test for categorical data, as appropriate. A *p* value <0.05 was considered significant. The analyses were performed using the Statistical Package for the Social Sciences (SPSS 22.0; SPSS Inc., Chicago, IL, USA). Significant trends over the years considered were assessed as average annual percent changes (AAPC), a summary measure of the trend over a given fixed interval that is computed as a weighted average of the annual percent change (APC) emerging from the joinpoint model, using weights equating to the length of the APC interval. If an AAPC lies entirely within a single joinpoint segment, the AAPC is the same as the APC for that segment [15].

## Results

**Fig. 1:**
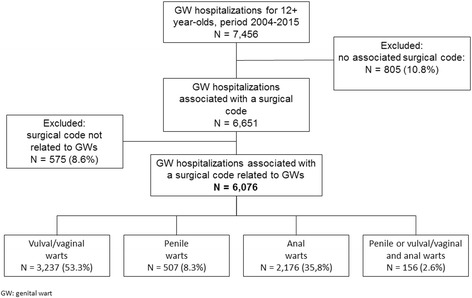
Flowchart of GW-related hospitalizations selected

From 1 January 2004 to 31 December 2015, there were 7456 hospitalizations associated with a diagnosis of condyloma in one of the six diagnosis fields, 6651 of these cases included a surgical code, which was potentially associated with a genital wart and could be grouped by genital anatomical site in 6076 cases (Fig. 1).

The 6076 cases eligible for our analysis included 3924 females (64.6%). The sample of patients as a whole was a median 32 years old (Interquartile Range [IQR] 25-42), the females 31 (IQR 25-40), and the males 35 (IQR 28-46). In 88.9% of cases they had been treated at a day hospital and the mean hospital stay was 1.6 ± 2.0 days, with no differences by gender or age group. Females were admitted to a gynecology ward (3093 patients) or to a general surgery ward (597 patients) in 94.0% of cases. Males were admitted to a general surgery ward in 73.7% of cases (1585 patients) and to a urology ward in 20.1% (432 patients). The remainder of the sample was admitted to other surgical wards. Overall, the ratio of vulval/vaginal to penile warts was 6.0:1, while the male-to-female ratio for anal warts was 2.5:1.

In the sample analyzed, 6076 patients received treatment: 3237 of the females (82.5%) were treated for vulval/vaginal warts, and 507 of the males (23.6%) for penile warts. An overall 2176 (35.8%) patients were treated for anal warts (75.4% of the males, and 14.1% of the females; *p* < 0.01); and 156 (2.6%) had combined treatments. Table 1 shows the patients’ characteristics by gender and type of treatment.

**Table 1:** Characteristics of the 6076 patients considered, by gender

Characteristics	Males		Females		Total	
	*n*	(%)	*n*	(%)	*n*	(%)
Age group						
12-20	65	(3.0)	356	(9.1)	421	(6.9)
21-27	468	(21.7)	1120	(28.5)	1588	(26.1)
28-47	1154	(53.6)	2035	(51.9)	3189	(52.5)
48+	465	(21.6)	413	(10.5)	878	(14.5)
Anatomical site						
Penile or vulval/vaginal	507	(23.6)	3237	(82.5)	3744	(61.6)
Anal	1623	(75.4)	553	(14.1)	2176	(35.8)
Penile or vulval/vaginal and anal	22	(1.0)	134	(3.4)	156	(2.6)

**Fig. 2:**
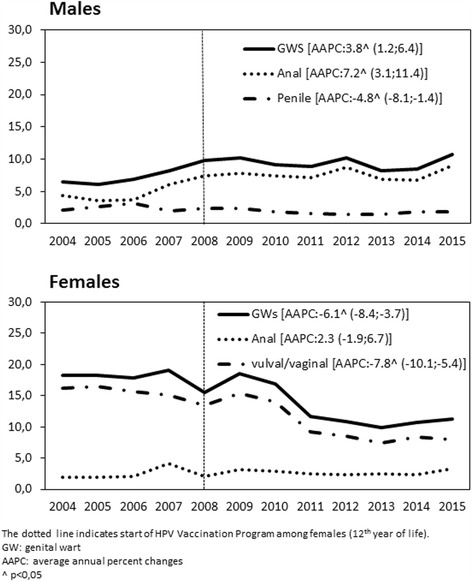
Overall GW-related hospitalization trends (× 100,000 population) in the Veneto region (2004-2015), by gender and anatomical site

The overall annual GW-related hospitalization rate during the study period was 11.8 per 100,000 population (8.6/100,000 males, and 14.8/100,000 females). The rate was stable among females up until 2007, with 19.1 hospitalizations per 100,000 in 2007, then fell significantly, reaching 11.3 per 100,000 hospitalizations for GWs in 2015 (AAPC: -6.1%; 95% CI: -8.4; −3.7). The overall trend of GW-related hospitalizations rose significantly in males over the study period, starting from 6.4 per 100,000 in 2004 and peaking in 2015 at 10.8 per 100,000 (AAPC: 3.8%; 95% CI: 1.2; 6.4). Figure 2 shows the GW-related hospitalization rate by gender and anatomical site, revealing a significantly declining trend for vulval/vaginal and penile warts, (AAPC: -7.8%; 95% CI: -10.1; −5.4 for vulval/vaginal warts; and AAPC: −4.8%; 95% CI: -8.1; −1.4 for penile warts).

**Fig. 3:**
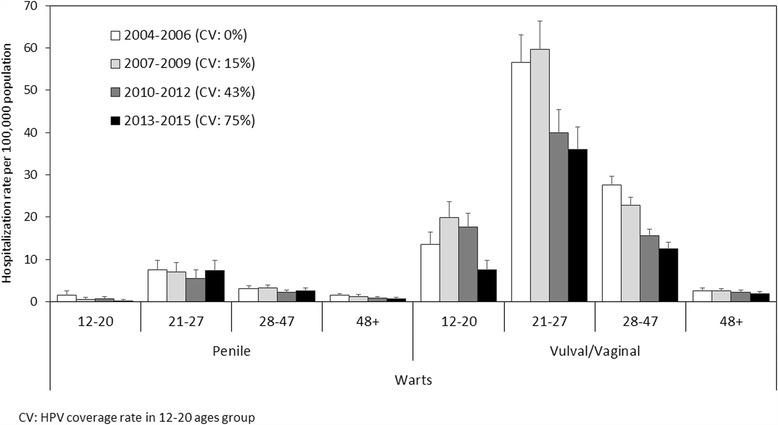
Average annual hospitalization rates for vulval/vaginal and penile warts by period, and by age group

Overall, the differences in the GW-related hospitalization rates for males between the periods 2004-2006 and 2007-2009, and between the periods 2007-2009 and 2013-2015 were 46.3% and −2.1%, respectively. Among females, the rate changed over the same periods by −0.7% and −41.0%, respectively (Fig. 3).

Among the 12- to 20-year-olds (the age group eligible for HPV vaccination according to our program), the hospitalization rate for vulval/vaginal warts dropped from 19.9/100,000 in the early vaccination period (2007-2009) to 7.6/100,000 in the last study period (2013-2015), with a decrease of 62.1%. For penile warts in the same age group, there was an even greater drop from 2007 to 2009 to 2013-2015 (−68.2%) (Fig. 3).

**Fig. 4:**
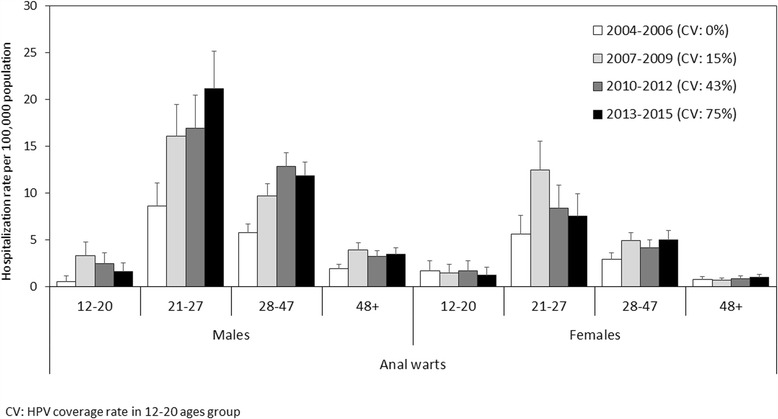
Average annual hospitalization rates for anal warts by study period, age group and gender

Figure 4 shows the rates anal warts by gender and study period; the highest hospitalization rate for anal warts concerned the 21- to 27-year-olds of both genders.

## Discussion

This study examined the trends of the hospitalization rates for GWs in a cohort of 5 million citizens and the results show that the burden of HPV-related disease on hospital resources is an important public health issue. It is not easy to estimate the morbidity of HPV disease in the general population due to differences in study design and population profiles, access to health care, and clinical data recording methods, especially for benign conditions.

In our sample, the overall hospitalization rate was 11.8/100,000 population per year, and it was 1.7 times higher for females than for males. On analyzing the different periods of the study, however, the hospitalization rate by gender was much the same in 2015, indicating a marked reduction in the hospital admissions involving females and an increase in those for males.

A previous work on HPV-related hospitalization in the same geographical area showed that hospital admissions for GWs increased in both genders from 2000 to 2010, pointing to the need for prevention programs [16]. The data emerging from the present study show a reversal of the trend of hospital admissions for the treatment of GWs, a number of factors might explain this picture besides the increase in HPV vaccination coverage rate. First, the hospitals (and gynecology departments in particular) in the Veneto region were reorganized in 2008; second, changes in people’s socio-economic status may theoretically have prompted some to go to private hospitals; third, changes in healthcare-seeking behavior or clinical practices [9] may have led to a greater use of self-applied topical treatments for genital warts instead of going to hospital.

The treatment strategy involves removing visible lesions until the host’s immune system can control viral replication. Several methods are available for the treatment of external GWs, and the choice of therapy depends largely on their morphology and extent. Common treatments include topical applications as well as surgical and obliterative approaches. Small GWs are managed mainly at primary care services or sexual health clinics, but larger lesions often need to be managed in the operating room by anogenital surgeons or gynecologists [17].

During the study period, there were fewer hospitalizations for penile warts than for vulvar/vaginal warts, and this may reflect a tendency for the former to be treated outside the hospital, since other studies found that ablative treatments were more often provided outside the hospital for men, whereas such procedures in woman required hospitalization [18, 19].

Our findings show a significant reduction over time in the hospitalizations for vulvar/vaginal warts, especially in the latter period (2013-2015), for patients from 12 to 20 years old, the age group for which the overall HPV vaccination coverage had reached about 75%. An example of the effectiveness of vaccination was seen in Australia, where the implementation of a vaccination program with 4HPV vaccine led to the decline and nearly to the disappearance of GWs in both men and women <21 years old [20, 21], and the same trend was seen for other, more complete surveillance systems [22]. Among the males in our sample (who had yet to be included in our regional vaccination strategy), the hospitalization rate for penile warts decreased among 12- to 20-year-olds, possibly suggesting a herd immunity [23].

On the other hand, the age- and gender-specific patterns in the declining numbers of penile and vaginal/vulva warts was not seen in the case of anal warts. This may have to do with differences in sexual practices, including the possibility of bisexuality and homosexuality (especially among males). Men who have sex with men (MSM) are a major risk group for sexually-transmitted diseases, and they have a high prevalence of anal warts due both to a greater susceptibility of the anal mucosa by comparison with the epithelium of the penis, and to a longer duration of anal HPV infections [24, 25]. The risk of anal HPV infection is 4-10 times higher among MSM than for heterosexual men. In addition to vaccinating this higher-risk group, it is very important to make more effort in terms of behavioral interventions to reduce the HPV infection rate in MSM [26–28].

It will be interesting to see if the male vaccination program introduced in our area in 2015 has any effect on the prevalence of GWs among males, including MSM, in future.

Our study has some limitations. For a start, we should consider the risk of the total number of HPV cases identified being inaccurate due to coding errors in the hospital records, although checking for the presence of relevant surgical codes should contain this selection bias. Another limitation lies in that the diagnoses could not be verified due to the lack of cytological and virological data. Finally, HDRs are unable to collect data that might have enabled us to ascertain the sexual practices of the study population.

## Conclusions

Our data suggest an early decline in the rate of GW-related hospital admissions in a population with a good HPV vaccination coverage. Routinely-recorded clinical data can be very useful for monitoring the impact of vaccination programs, but further efforts should be made to establish methods for better estimating the epidemiological picture regarding HPV-related diseases with particular regard to sexual behavioral.

## Abbreviations

AAPC: Average annual percent changes
APC: Annual percent change
GW: Genital warts
HDR: Hospital discharge records
HPV: Human papillomavirus
ICD-9-CM: International Classification of Diseases, Ninth Revision, Clinical Modification
IQR: Interquartile range
MSM: Men who have sex with men
